# A2B5 Expression in Central Nervous System and Gliomas

**DOI:** 10.3390/ijms23094670

**Published:** 2022-04-23

**Authors:** Dominique Figarella-Branger, Carole Colin, Nathalie Baeza-Kallee, Aurélie Tchoghandjian

**Affiliations:** 1 Aix-Marseille University, CNRS, INP, Inst. Neurophysiopathol, 13005 Marseille, France; carole.colin@univ-amu.fr (C.C.); nathalie.baeza@univ-amu.fr (N.B.-K.); aurelie.tchoghandjian@univ-amu.fr (A.T.); 2 APHM, CHU Timone, Service d’Anatomie Pathologique et de Neuropathologie, 13005 Marseille, France

**Keywords:** ganglioside, A2B5 IgM, central nervous system, glioma, cancer stem cell

## Abstract

A2B5 IgM recognizes c-series gangliosides with three sialic acids. The aim of this review was to focus on A2B5 expression in the central nervous system and gliomas. In brain development, A2B5+ cells are recorded in areas containing multipotent neural stem cells (NSC). In adults, A2B5+ cells persist in neurogenic areas and in white matter where it identifies oligodendrocyte precursor cells (OPCs) but also cells with NSC properties. Although the expression of A2B5 has been widely studied in culture, where it characterizes bipotential glial progenitor cells, its expression in vivo is less characterized mainly because of technical issues. A new interest was given to the NSCs and OPCs since the discovery of cancer stem cells (CSC) in gliomas. Among other cell surface molecules, A2B5 has been identified as an accurate marker to identify glioma CSCs. We and others have shown that all types of gliomas express A2B5, and that only A2B5+ cells, and not A2B5- cells, can generate a tumor after orthotopic implantation in immunocompromised animals. Moreover, A2B5 epitope expression is positively correlated with stemness and tumor growth. This review highlights that A2B5 is an attractive target to tackle glioma CSCs, and a better characterization of its expression in the developing and adult CNS will benefit to a better understanding of gliomagenesis.

## 1. Gangliosides Recognized by the A2B5 Antibody

### 1.1. Generation of the A2B5 Monoclonal Antibody

In 1979, a monoclonal antibody recognizing an antigen of neurons was reported by Eisenbarth et al. [1]. This antibody was produced by hybrid cells formed by the fusion of mouse myeloma cells with spleen cells from mice immunized with 8-day chicken embryo retina cells. In chickens, this antibody is bound to neural tissue such as the retina, brain, spinal cord and dorsal root ganglia but not to muscle, heart, kidney or liver cells. The antibody also recognized the bovine and human brain. The antibody recognized the external surface of neuron cells bodies. The antibody was cytotoxic to 8-day embryo-retina cells. The A2B5 antigenicity was not destroyed by trypsin or by incubation at 100 °C but was lost after treatment with *Clostridium Perfringens* neuraminidase. This suggested that the A2B5 antibody recognizes a target that has properties of a glycolipid, and further experiments favored that it was a complex ganglioside containing at least three sialic acids.

### 1.2. Structure, Biosynthesis and Expression of Gangliosides 

**Figure 1:**
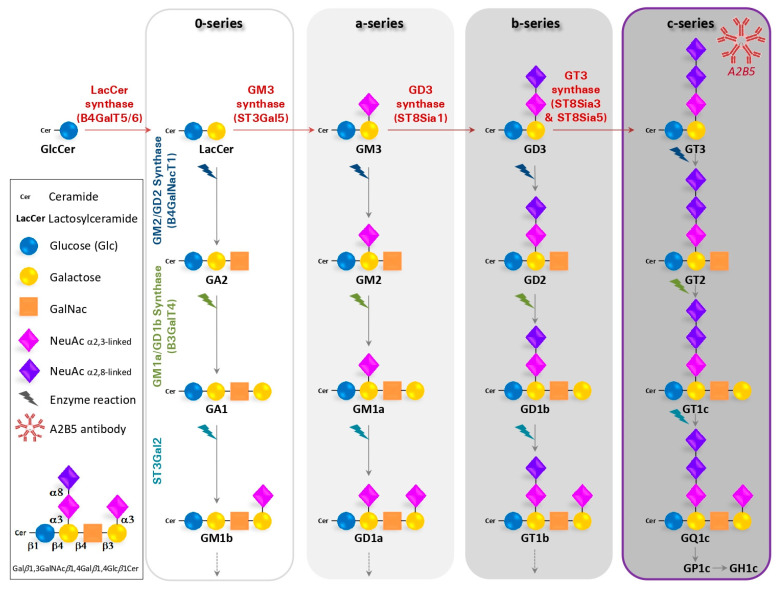
Schematic biosynthesis pathway for gangliosides.

Gangliosides were first described in 1935 while studying tissues from patients suffering of a Niemann–Pick disease [2]. They are glycosphingolipids composed of a ceramide lipid tail attached through glycosidic linkage to a glycan headgroup containing one or more sialic acid residues (Figure 1). The term “sialic acid” is used to refer to any member of the neuraminic acid family (comprising N-acetyl and N-glycolyl derivatives of neuraminic acid, respectively, NeuAc and NeuGc) (reviewed in [3]). In cells, gangliosides are primarily, but not exclusively, localized in the outer leaflets of plasma membrane, within specific cell surface microdomains termed lipid rafts [4]. The hydrophobic ceramide tail inserts into the outer phospholipid leaflet, while the glycan head group extends outwardly and engages interactions in *cis* (within the same membrane) as well as in *trans* (with molecules on other cells and in the extracellular space), leading to the modulation of cell signaling and cell–to–cell communication [5]. 

Gangliosides are usually named according to the system suggested by Svennerholm [6]. This nomenclature was initially based on the migration order of gangliosides in chromatography. The core structure of neutral sugars defines the name of a respective series, in which the pyranose forms *N*-Acetylgalactosamine (GalNAc) are attached in a defined order and linkage to lactosylceramide (Gal*β*1,4Glc*β*1Cer). The names contain information about the series (“G” = ganglio) and the number of sialic acids (“A” = 0, “M” =1, “D” = 2, “T” = 3, “Q” = 4, ...). For example, GM1 is defined by the sequence Gal*β*1,3GalNAc*β*1,4Gal*β*1,4Glc*β*1Cer. Sialic acids (NeuAc in human) can be attached once, twice, or several folds to different positions within the core structures. Most often, they are found in *α*2,3-linkage to the “inner” or “outer” galactosyl residue and in *α*2,8-linkage to other sialic acids. Moreover, the outer sialic acid residue can be further *O*-acetylated, *N*-acetylated or sulfated [7]. In general, gangliosides of the 0-series bear no sialic acids on the galactose, whereas gangliosides of the a-, b- and c- series bear one, two and three sialic acid residues, respectively. 

Gangliosides are primarily synthetized in the endoplasmic reticulum and further modified in the Golgi apparatus by the sequential addition of additional carbohydrate moieties to an existing acceptor lipid molecule. It starts by the transfer of sialic acid residues to lactosylceramide (LacCer) by specific sialyltransferases, i.e., the CMP-Neu5Ac: LacCer α2,3-sialyltransferase ST3Gal V (GM3 synthase) [8], the CMP-Neu5Ac: GM3 α2,8-sialyltransferase ST8Sia I (GD3 synthase) [9] and the CMP-Neu5Ac: GD3 α2,8-sialyltransferase ST8Sia V (GT3 synthase), that show high specificity toward glycolipid substrates [10]. The ST8-a-N-acetyl-neuraminide α-2,8-sialyltransferase 3 (ST8SIA3) is highly expressed in brain and mediates the sialylation of a diversity of glycolipids including GD3 as well as selected glycoproteins. Thus, LacCer, GM3, GD3 and GT3 are the precursors for 0, a-, b- and c-series gangliosides, respectively (Figure 1). Then, the disaccharide core Galβ1-4Glcβ can be elongated by the sequential action of the N-acetylgalactosaminyltransferase (β4GalNAc T1, also called GM2/GD2 synthase [11], the β1,3-galactosyltransferase 4 (β3Gal T4 or GMa/GD1b synthase) [12] and by sialyltransferases (ST3Gal II and ST8Sia V)). Furthermore, sialic acids are removed by the enzymatic action of sialidases, also called neuraminidases. In humans, they are encoded by the genes Neu1 to Neu4 [3,13].

Total mass and specific ganglioside composition vary significantly from tissue to tissue and among different cell types and also according to development stages. The human brain contains 10- to 30-fold more gangliosides than any other tissue or organ in the body. Moreover, gangliosides account for 80% of all glycans and >75% of the sialic acid present in the brain, whereas in other organs, >80% of glycans and sialic acid residues are linked to glycoproteins (reviewed in [14]). In the rodent and human brain, the ganglioside profile is dominated by simple gangliosides (GM3 and GD3) during development, whereas at later developmental stages and in adults, complex gangliosides (GM1, GD1a, GD1b and GT1b gangliosides) become predominant [15].

The gangliosides’ biosynthesis involves sequential activities of distinct glycosyltransferases and sialyltransferases, as illustrated for the main 0- (asialo), a-, b- and c-series of gangliosides. Gangliosides are synthesized by the stepwise addition of monosaccharides (Glucose-Glc, Galactose) to Ceramide (Cer). The extension of GlcCer to make LactosylCeramide (LacCer) occurs through the action of the Lactosyl-ceramide synthase (B4GalT5/6). Then, the GM3 synthase (ST3Gal5) transfers a first sialic acid residue to form GM3. Subsequently, GM3, GD3 and GT3 serve as precursors of an increasingly heterogeneous range of complex gangliosides. GA refers to asialo-ganglioside while GM, GD, GT and GQ are named according to the increasing number of sialic acid residues added to the galactose.

### 1.3. The A2B5 Monoclonal Antibody Recognizes C-Series Gangliosides

Several studies have reported that the A2B5 antibody, which is of IgM-type, recognizes c-series gangliosides, mainly GT3 and its 9-*O*-acetylated form and, to a lesser extent, GQ1c [16,17,18]. Furthermore, Saito et al. [18] purified gangliosides from bonito fish brain and demonstrated that the A2B5 reactivity from c-series gangliosides decreased in the order of GT3 > GQ1C > GP1C > GH1c. Moreover, the A2B5 antibody did not react with GD3 and other gangliosides and neutral glycosphingolipids. Based on these results, it was concluded that the antigenic epitope for A2B5 included a trisialosyl residue linked to the inner galactose of the hemato- or ganglio-type oligosaccharide of c-series gangliosides. Moreover, Inoko et al. [19] showed in mice that the α-2-8 trisialic acid epitope recognized by A2B5 resides not only on gangliosides but also on glycoproteins during development, although this finding has not been confirmed.

## 2. A2B5 Expression in the Vertebrate Central Nervous System 

### 2.1. The Origin of Neurons and Glia 

**Figure 2:**
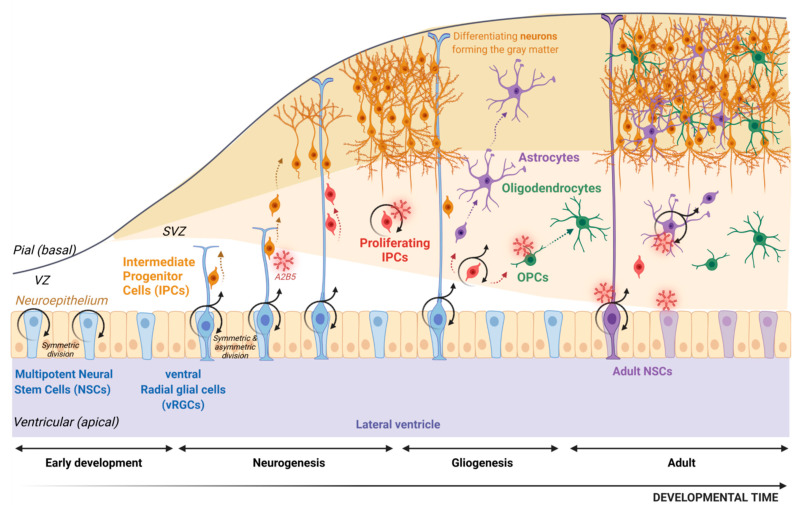
Schematic representation of neurogenesis and gliogenesis processes during development and in adulthood.

During the embryonic development of the vertebrate nervous system, neurons and glia (including astrocytes, oligodendrocytes and ependymal cells) derive from multipotent neural stem cells (NSCs) located in the neuroepithelium (Figure 2). These NSCs undergo symmetric division that expands the neuroepithelial surface area and therefore the pool of NSCs, although some can generate neurons. Then around E9–E10 in mice, the NSCs are transformed into radial glia cells which line the forebrain ventricles and the spinal canal. In the forebrain, because of their location in the ventral telencephalon, they are referred as ventral radial glia (vRG). vRG are now considered as NSCs [20]. The cell bodies of vRG are confined to the ventricular zone, but their processes span the width of the developing spinal cord or forebrain. One major function of vRG is to serve as guides for the radial migration of neural cells from proliferative zones to their post-mitotic destination [21]. In this review, we will use the terminology NSC for the multipotent cells able to initiate both neurons and glial cells. vRG cells first maintain their numbers by proliferating through symmetric division. When they undergo asymmetric division, they generate a daughter cell that is either a neuron or a restricted intermediate progenitor cell (IPC). IPCs, also called “transient amplifying cells”, are restricted in their fate (reviewed in [22]) and give rise to neurons or glial cells [23,24,25]. Neurogenesis and gliogenesis are temporally segregated in the developing rodent neocortex and spinal cord, neurogenesis preceding gliogenesis [24].

In the developing human brain, a distinct anatomical zone called the outer subventricular zone (O-SVZ) is observed starting at the 12th post-conception week. It is formed by proliferative IPCs recorded as outer radial glia (oRG) [26,27,28,29], some of them deriving from vRG. Most vRGs convert into astrocytes at the end of development [30,31,32], but some remain postnatally where they function as NSCs in the adult. 

In the adult rodent brain, NSCs with the ability for long-term self-renewal and the generation of neurons and glia persist in the subventricular zone (SVZ, lining the lateral ventricles) and the subgranular zone (SGZ, within the dentate gyrus of the hippocampus) where they are located in specific niches. Importantly, NSCs in both SVZ and SGZ have astroglial properties [33,34]. In addition, a population of adult NSCs can be isolated from the subcortical white matter [35,36].

During the embryonic development of the vertebrate nervous system, neurons and glia derive from multipotent neural stem cells (NSCs) located in the neuroepithelium. These NSCs undergo symmetric division; then, they differentiate into radial glia cells referred to as ventral radial glia (vRG). Their processes span the width of the developing forebrain and serve as guides for the radial migration of intermediate progenitor cells (IPCs) from proliferative zones to their post mitotic destination (grey matter for neurons, subventricular zone and SVZ and grey matter for astrocytes and oligodendrocytes). Indeed, vRG proliferate through symmetric division, but they can also go through asymmetric division and generate a daughter cell that is either a neuron or restricted proliferating IPCs with a glial fate (astrocyte precursor cell or oligodendrocyte precursor cell, OPC). Although most vRGs convert into astrocytes at the end of development, some remain in adulthood where they function as NSCs in adults. Through development and in the adult, the A2B5 marker is shared by several cell types as restricted IPCs, proliferating IPCs, OPCs and adult NSCs.

### 2.2. The Oligodendroglial Lineage 

Oligodendroglia, the most prevalent cell lineage within the white matter, includes oligodendrocyte precursor cells (OPCs, also referred in some papers as oligodendrocyte progenitors – OLPs), immature pre-myelinating oligodendrocytes and mature myelinating oligodendrocytes. During both embryonic development and within neurogenic niches in adults, the majority of OPCs are specified from NSCs; they likely correspond to IPCs that are committed to the oligodendroglial lineage. Major insights into the oligodendrocyte development have been gain from animal studies. Briefly, in development, OPCs of the cerebral hemispheres, cerebellum and spinal cord are derived from committed NSCs that generate three sequential waves. For each wave, NSCs located in discrete domains of the SVZ are specified into OPCs that express a combination of transcription factors under the control of external clues including Sonic Hedgehog (SHH), bone morphogenic proteins (BMP) and fibroblast growth factors (FGF) (reviewed in [37,38,39]). As an example, in the rodent developing a forebrain, the first generation of OPCs originates from NSCs located in the SVZ, in a restricted region spanning the boundary between the anterior hypothalamus (diencephalon) and the medial ganglionic eminence (telencephalon) at embryonic day E12.5, under the transcriptional control of Nkx2.1. This region has been called the “anterior entopedoncular area” [40]. The second wave occurs few days later from NSCs located in the lateral ganglionic eminence under the transcriptional control of Gsh2 [41]. The last wave occurs just before birth from dorsal NSCs committed to OPCs under the transcriptional control of Emx1 [42]. It appears that at each wave, OPCs proliferate then migrate in the parenchyma to achieve a relatively uniform distribution in white and gray matter. When they reach their final destination, most of them differentiate into mature oligodendrocytes whose main function is to produce myelin, whereas some persist as resident OPCs representing up to 3 or 5% of all cells in the adult mouse brain [43,44]. Although these resident OPCs are maintained at an immature, slowly proliferative or quiescent state in the adult central nervous system (CNS), they are still able to proliferate in response to local signals, giving rise predominantly to oligodendrocytes [45]. Importantly, progenitors cells of the oligodendrocyte lineage with different temporal and spatial origins in the murine CNS converge into a similar OPC transcriptional stage at post-natal day 7 (P7) [38].

Although the generation of OPCs appears to be conserved in humans, the final oligodendrocyte population is much more numerous than in their rodent counterpart. In humans, the O-SVZ contains EGFR-expressing pre-OPCs, which define an additional pool of OPCs able to produce a large number of oligodendrocytes [46]. In addition to resident OPCs, OPCs can still be generated in adults from neurogenic niches, mainly SVZ [47]. Although it seems that in vivo the lineages of oligodendrocytes and astrocytes appear to be segregated early, the fate of OPCs in vitro differs since OPCs are able to generate oligodendrocytes and type-2 astrocytes [48,49,50]. A common oligodendrocyte-astrocyte bipotential glial progenitor has not been found in vivo yet [51,52] (reviewed in [39]). 

### 2.3. A2B5 Expression Characterizes the Bipotential Glial Progenitor Cells (O-2A) In Vitro 

**Figure 3:**
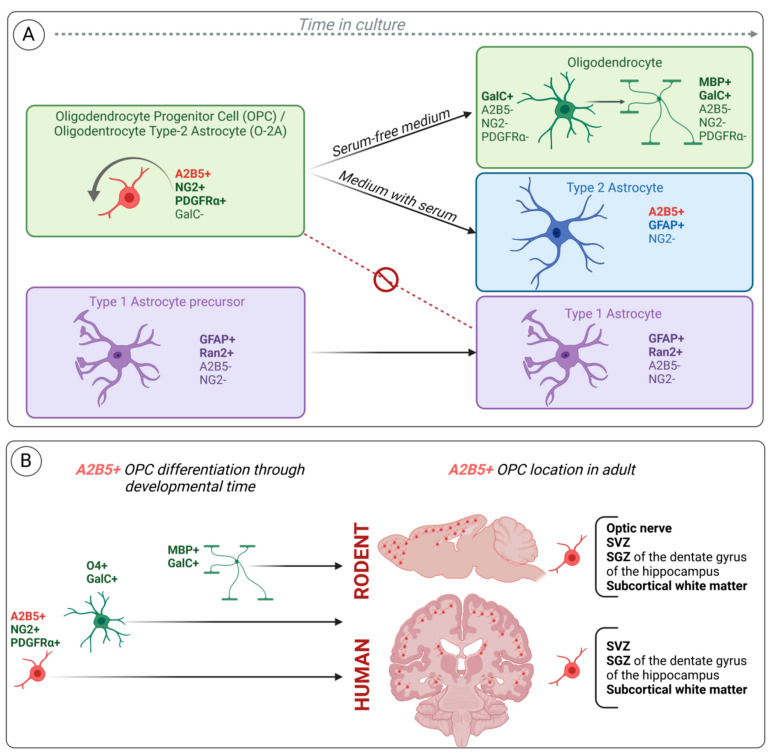
Schematic representation of the oligodendroglial lineage markers highlighted in vitro (**A**). A2B5+ OPC differentiation through developmental time and its location in adult (**B**). (**A**) Oligodendrocyte precursor cells (OPC), also known as oligodendrocyte type-2 astrocyte progenitor cells (O-2A), are characterized by A2B5, NG2 and PDGFRα expression. In serum-free medium, A2B5+ OPCs/O-2A differentiate into GalC+ oligodendrocytes while losing A2B5, NG2 and PDGFRα as they differentiate. These oligodendrocytes can further differentiate to expressing myelin basic protein (MBP), but they never achieve a full mature myelinating oligodendrocyte phenotype. In the presence of serum, OPCs/O-2A differentiate into type-2 astrocytes characterized by A2B5 and GFAP co-expression. However, type-1 astrocyte precursors defined by the lack of A2B5 are not generated from OPC/O2A and differentiate into A2B5- GFAP+ type-1 astrocyte. They represent a different lineage. (**B**) A2B5+ OPCs/O-2A differentiate through developmental time into immature (O4+, GalC+) and mature (MBP+, GalC+) oligodendrocytes while losing A2B5 marker. Nevertheless, in adulthood, A2B5+ OPCs/O-2A persist in specific neurogenic areas and subcortical white matter of rodent and human brains. SVZ: subventricular zone; SGZ subgranular zone.

The mouse A2B5 monoclonal antibody was initially found to recognize a ganglioside on neuronal cells [1]. Subsequently, this antibody was used in culture from neonatal optic nerves containing glial cells but no neurons. In this in vitro model, the antibody labelled cycling bipolar cells as well as stellate cells, both cell types expressing, in addition to A2B5, NG2 and PDGFRα *(*Platelet-derived growth factor receptor α*)* but not GFAP [48,49,50,53,54]. When these dividing A2B5 expressing-cells were placed in serum-free medium, they were able to differentiate into oligodendrocytes characterized by multiple cell processes and galactocerebroside expression but neither A2B5 nor NG2. In contrast, in the presence of fetal calf serum and BMP or CNTF, the dividing A2B5-expressing cells generated type-2 astrocytes characterized by multiple cell processes and GFAP and A2B5 co-expression. The type-2 astrocyte differs from the so-called type-1 astrocyte, which is characterized by a flat morphology and GFAP without A2B5 expression. This cell type is not derived from A2B5+ cells and forms a distinct lineage. Therefore, A2B5+ cells represent bipotential glial progenitors and thus were named O-2A (oligodendrocyte/type-2 astrocyte) progenitor cells (Figure 3A). 

Bipotential A2B5+ cells have also been isolated from the adult rat optic nerve [55,56]. In contrast to what was observed in optic nerve, Raff et al. [49] failed to identify type-2 astrocytes in cultures from subpial cortical grey matter or cerebellar cortex. Moreover, by using the A2B5 antibody on tissue sections, it was shown that fibrous astrocytes in the adult optic nerve were immunostained in contrast to protoplasmic astrocytes in the cerebral cortex [57].

### 2.4. A2B5 and Other OPC Cell Surface Markers: NG2 and PDGFRα 

Although A2B5 was the first-discovered OPC cell surface marker shown to be highly relevant for labelling cells in culture, it appears that it was technically difficult to use it for immunohistochemical detection in routinely processed tissue section. Because OPCs proliferate in response to PDGFα, it was subsequently demonstrated that OPCs express PDGFRα [58,59], this marker being subsequently used to characterize OPCs in vivo [60]. In addition, another study reported that bipotential glial precursor cells in the optic nerve express the NG2 proteoglycan [61]. A careful comparison of PDGFRα and NG2 expression by double immunofluorescence labelling demonstrated a complete overlap between glial cells that expressed NG2 and those that expressed PDGFRα both in vitro and in the developing rat brain in vivo [62,63]. OPCs are characterized by a bipolar morphology and a combination of cell surface markers, including NG2, PDGFRα and A2B5, and transcriptional markers such as OLIG1 and 2, MYT1, NKX2.2, NKX2.6, SOX9 and SOX10 [64,65,66] (reviewed in [37,67]). Although most of the transcription factors expression is maintained in pre-myelinating and myelinating oligodendrocytes, the cell surface markers change as the cell differentiate (reviewed in [37,67,68]). However, PDGFRα and NG2 are not specific to OPCs: PDGFRα is expressed on embryonic neurons, and NG2 is expressed on vascular mural cells in the CNS as well as on immature progenitor cells of mesenchymal lineage and in microglia [39,69].

Unfortunately, no systematic comparison between A2B5, NG2 and PDGFRα expression has been conducted in vivo likely because of the difficulties to obtain nice immunostainings, and A2B5 expression is rarely found in the literature since the use of NG2 and PDGFRα as OPC markers. However, Baracskay et al. [70] have shown that in the culture of the developing brain, NG2+ cells and A2B5+ cells arise from overlapping cell populations. NG2+ cells appear prior to the expression of A2B5+ cells and generate A2B5+ cells. Therefore NG2+/A2B5- cells can be considered pre-OPCs.

There are, however, some studies focusing on cells expressing these markers but they mainly concern in vitro experiments. As an example, Zhou et al. [71] have characterized A2B5+, A2B5-, NG2+ and NG2- cells obtained from NSCs derived from the human fetal brain after in vitro differentiation into OPCs. In the whole cell population, flow cytometry demonstrated that PDGFRα expression was recorded on up to 75% of the cells, whereas A2B5+ cells accounted for approximately 26% and NG2 for 15%. Importantly, the proportion of PDGFRα expressing cells was higher in NG2- or A2B5- cells in comparison to their positive counterparts. Each of these cell subsets had distinct proliferating, migrating and myelination ability. Unfortunately, corroborating these data by a study on human OPCs in vivo would be technically difficult. 

### 2.5. A2B5 Expression Characterizes Glial-Restricted Precursor Cells Derived from Multipotent NSCs

The A2B5 antibody was also used to demonstrate that glial-restricted precursor cells (GRPs) are derived from multipotent NSCs [72]. NSCs in vivo and in vitro isolated from E10.5 rat embryos platted on fibronectin/laminin substrate and cultured under non-differentiation conditions (bFGF and chick embryonic extract) do not express any glial markers. However, in the absence of chick embryonic extract, NSCs give rise to self-renewing A2B5+ cells that are able to differentiate into oligodendrocytes and astrocytes but fail to differentiate into neurons under conditions that promote neuronal differentiation in NSCs. This study showed that A2B5+ cells are glial IPCs derived from multipotent NSCs. 

### 2.6. A Subset of Radial Glia Cells in Rodent Expresses A2B5 during Development and in Adulthood

The A2B5 antibody was instrumental to demonstrating that the spatially restricted subpopulation of radial glia along the dorsal–ventral axis in forebrains of E15.5 rat embryos acquires different markers for neuronal or glial precursors during CNS development [73]. In this study the authors analyzed the expression of a combination of three cell-type specific markers: BLBP (brain–lipid-binding protein) for radial glia, 5A5/E-NCAM (Embryonic–Neural Cell-Adhesion Molecule) for neuronal precursors and A2B5 for glial precursors both on cortical radial glia in vivo and their progeny in vitro. They observed that in vivo BLBP expression precedes the expression of the other markers and that A2B5 expression is spatially restricted and co-localized with a subset of BLBP cells. A2B5-labelled cells are mainly in the lateral part of the forebrain extending into the lateral ganglionic eminence (LGE), and confocal microscopy confirmed its overlap on radial glia: A2B5+ cells overlap with BLBP+ radial glia in more ventral parts of the cortex and in the VZ of the LGE. Moreover, immunostaining on cells dissociated from E15.5 embryonic forebrains demonstrated that 70% of cells dissociated from LGE co-expressed BLBP and A2B5, whereas the co-expression was observed in 27.8% of the cells from the cortex. Another study reported the co-expression of A2B5 and GLAST, another radial glia marker in postnatal mouse brain [74]. In this study, cortical hemispheres or cerebellums of P1, P3 and P7 mice were dissociated, and the expressions of cell surface markers including A2B5 and GLAST were investigated by flow cytometry. Moreover, 3.5–4.5% of A2B5+/GLAST+ cells were recorded in the cortical hemispheres and cerebellums of P1 mice, while 9–22% GLAST single-positive and 8–14% A2B5 single-positive were detected. The percentage of double positive cells decreased to 0.5–2% at P7. Taken together, these two studies demonstrated that the A2B5 antibody recognizes a subset of radial glia cells both during development and in adult rodents, and that A2B5 expression is not restricted to cells committed to the oligodendrocyte lineage (Figure 3B).

### 2.7. A2B5 Identifies OPCs and Multipotent Neural Progenitor Cells from Subcortical White Matter of the Adult Human Brain

The identification, isolation and characterization of human OPCs is of utmost importance to understanding the biology of these cells and to use them as therapeutic approaches for demyelinating diseases. Although previous studies reported the existence of cycling OPCs in adult rodent subcortical white matter [43], Roy et al. [75] were able to isolate and characterize OPCs from adult human subcortical white matter. To this aim, they dissociated cells from surgical adult human brain tissue (obtained from epilepsy surgery) and labelled them with different antibodies including A2B5. They observed bipolar A2B5+ cells after 4 days in culture. Furthermore, the authors transfected white matter dissociated cells with hGFP (humanized gene for green fluorescent protein) placed under the control of the promoter for 2′,3′-cyclic nucleotide 3-phosphodiesterase (CNP) (pCNP2:hGFP+), CNP being the earliest myelin-associated protein synthesized in developing oligodendrocytes [76]. They observed at 6–13 days post-transfection that up to 60% GFP+ cells were bipolar and expressed A2B5+. After 4 weeks, most of the cells expressed the oligodendroglial marker O4. This indicates that adult human white matter contains a population of mitotically competent OPCs (Figure 3B).

A few years later, it was demonstrated that in low-density cell culture, human white matter progenitor cells (WMPCs) were able to generate not only oligodendrocytes but also neurons [36]. In this study, the authors showed that A2B5 cell surface marker can be used as a surrogate marker for pCNP2:hGFP+ cells, and thus they used magnetic activating cell sorting (MACs) to isolate A2B5+ cells from human adult white matter cells. The incidence of A2B5+ sorted cells was 3.6 +/- 0.3%. A fraction of A2B5+ cells were clonogenic (able to generate spheres in a limit-dilution assay) and were multipotent in vitro although they showed a limited self-renewal capability when primary spheres were dissociated to form secondary spheres. In contrast, A2B5- cells were unable to generate spheres. At last, when the A2B5+ cells isolated from human white matter were transplanted into a E17 fetal rat brain, they were able to integrate into the rat forebrain SVZ as neuronal progenitors and generate granule and striatal neurons. This important study demonstrates that a pool of mitotically competent neurogenic progenitor cells is located in the adult human white matter, and these cells express the A2B5 antigen (Figure 3B).

In another study, it was shown that human adult WMPCs have the same multipotent capacities as adult hippocampal progenitors [77]. In this study, freshly isolated cells from subcortical white matter and the hippocampus were shown to express A2B5 and NG2 in around 20% of cells, although NSC markers such as CD133, nestin, Sox2 or PAX6 were not observed. Double labelling reveals 4.9%–9.8% A2B5+/NG2+ co-expressing cells with no difference between white matter and hippocampal cells. A small fraction of cells (1 out of 694 and 1 out of 1331 from white matter and hippocampal cells, respectively) were able to generate neurospheres with multipotent differentiation ability in culture. Whole transcriptome analyses showed strong similarities between adult white matter progenitor cells and adult hippocampal progenitors [77]. Taken together, these results further demonstrate that human adult white matter contains up to 20% of cells expressing A2B5 and that human white matter cells have NSC properties. 

## 3. A2B5 Expression and Glioma

### 3.1. Human Glioma: WHO Classification and Cell of Origin 

Gliomas are the most common primary intracranial neoplasms in humans. Gliomas are classified according to the World Health Organization (WHO) of CNS tumors. Since its first edition, this classification has been revised few times, leading to major changes in glioma classification. This has to be taken into account when publications include glioma samples that are always classified according to the latest WHO classification at the time of the study. As an example, mixed gliomas called oligoastrocytoma were no longer recognized as tumor-type in the 2016 WHO classification, which included molecular markers for the first time. This group of tumors was no longer accepted because diffuse grade II and III adult gliomas displayed molecular features of astrocytomas (*IDH* mutation associated with *TP53* or *ATRX* mutation without 1p/19q co-deletion) or oligodendroglioma molecular markers (*IDH* mutation and 1p/19q co-deletion) [78]. Moreover, in the 2016 WHO classification, two glioblastomas (GBM, grade IV) types emerged: glioblastoma, *IDH*-mutant and glioblastoma, *IDH*-wild type. GBM, *IDH*-mutant was no longer recognized in the 2021 WHO classification, and by definition, a GBM in an adult is always *IDH*-wild type; in the subsequent paragraphs, this tumor type is referred to as GBM [79]. Besides, the 2021 WHO classification recommends using Arabic numbers instead of Roman to assess the grade. Fortunately, most studies focused on GBM have included “true” GBM, meaning *IDH* non-mutated tumors. GBM is the most frequent glioma in adults. The median overall survival of GBM does not exceed 15 months in spite of the current treatment, which nowadays still relies on surgery followed by radiotherapy plus concomitant and adjuvant temozolomide [80]. In the last 20 years, GBM tumor progression has been proposed to rely on cancer stem cells (CSCs) resulting from the malignant transformation of NSCs. Glioma CSCs were defined by the following properties: ability to proliferate and generate multipotent clones of cells called gliomaspheres in an appropriate medium; self-renewal capacity as demonstrated by subspheres-forming assay; differentiation along neural, astrocytic and oligodendroglial lineages in vitro; and tumorigenicity when implanted in immunodeficient animals, in vivo [81]. However, the cell of origin of gliomas remains a matter of debate, although a consensus arose that gliomas derive from transformed adult precursor cells, adult NSCs and OPCs being the major candidates (reviewed in [82]). In this context, A2B5 is a relevant biomarker to be searched for in gliomas. 

### 3.2. A2B5 Expression Is Observed in All Glioma Types

**Figure 4:**
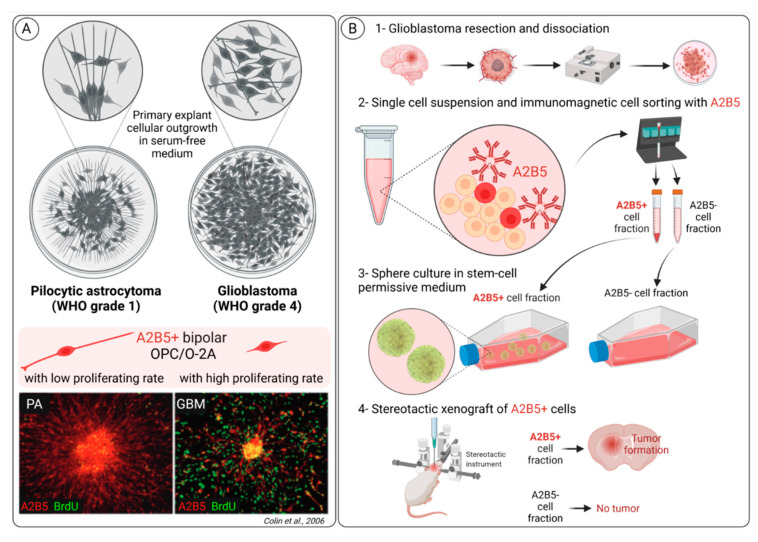
Overview of A2B5 expression in glioma highlighting A2B5 as a GBM CSC marker. (**A**) A2B5+ bipolar OPC/O-2A cells are observed in all glioma types but harbor distinct migration and proliferation capacities, depending on the WHO grade of glioma (pilocytic astrocytoma—PA, grade 1, versus glioblastoma—GBM, grade 4). In primary explant culture assays, A2B5+ cells from PA did not proliferate (few A2B5+/BrdU+ cells); they had a bipolar appearance with very long cell processes, one of them being in close contact with the explant. In contrast, A2B5+ cells from GBM migrated far away from the explant and were dividing (numerous A2B5+/BrdU+ cells). (**B**) We and others have generated A2B5+ cancer stem cell GBM cell lines; here we summarize our procedure, from (1) tumor resection and mechanic and enzymatic dissociation, (2) A2B5 immunomagnetic cell sorting (A2B5+ cell fraction was about 20% of the single cell suspension), (3) culture in a serum free stem-cell permissive medium to form gliomaspheres and (4) demonstration that only A2B5+ cell fraction gives rise to tumor development after stereotactic xenograft in mice.

Few years ago, by using an in vitro assay, we have identified and characterized glial precursor cells in various glioma types [83]. In this study, we performed primary explant cultures from a large series of glioma types classified according to the WHO 2007 as pilocytic astrocytoma grade I, diffuse adult gliomas grades II and III and GBM grade IV (Figure 4A) [84]. We observed in all glioma types at 1 day in culture the expression of A2B5 in bipolar cells. Importantly, the A2B5+ cells from different glioma types harbored distinct migration and proliferation capacities. A2B5+ cells from pilocytic astrocytoma did not proliferate; they had a bipolar appearance with very long cell processes, one of them being in close contact with the explant. In contrast, A2B5+ cells from GBM migrated far away from the explant and were dividing. We also observed that the differentiation capacities of the A2B5+ cells differed according to the histological types: A2B5+ cells from pilocytic astrocytoma, and to a lesser extent those isolated from GBM, had differentiation capacities reminiscent of normal O-2A cells in culture but never expressed MBP (myelin basic protein), a maker of mature oligodendrocytes. In contrast, A2B5+ cells isolated from grade II and III glioma demonstrated high proliferation rates but did not migrate nor differentiate in culture. 

This study corroborates a previous one showing the immunohistochemical expression of both NG2 and PDGFRα in various glioma types including pilocytic astrocytoma, GBM and other diffuse gliomas classified at this time as “oligodendroglioma “and “fibrillary astrocytoma” [85]. The expression was strong and obvious in pilocytic astrocytoma and oligodendroglioma but absent in 4/5 of the GBM studied. In keeping with our results, MBP expression was not detected. A2B5 expression was not searched for in this study. Other authors also reported concomitant expressions of A2B5, O4 and PDGFRα in a series of 25 gliomas of various types and grades. GFAP was also found but not MBP [86]. 

In another study [87], the quantification of A2B5-expressing cells was performed by both MACS and flow cytometry in a large series of infiltrative gliomas classified according to the relevant WHO classification of brain tumors at this time and including grade II gliomas (oligodendrogliomas, diffuse astrocytomas and mixed oligoastrocytomas), grade III anaplastic gliomas (oligodendrogliomas, astrocytomas and mixed oligoastrocytomas) and GBM. A2B5+ cells were recorded in all tumor samples and were not related to histological types or grade. However, the number of A2B5+ cells was higher in glioma samples (around 14%, n = 29) than in the cortex or white matter of surgical samples (n = 54) obtained from epilepsy surgery where A2B5+ cells did not exceed 3.6%. Moreover, a large number of A2B5+ cells from gliomas were mitotic, and A2B5+/KI67+ cells accounted for 31% of the A2B5+ cells. Interestingly, the telomerase activity of GBM-derived A2B5+ was high in contrast to low grade-derived A2B5+ cells or those derived from normal adult white matter [87]. 

Taken together, these studies showed that all gliomas contain cells expressing A2B5, a marker also shared by OPCs. However, these A2B5 cells exhibit some differences in their properties (morphology, proliferation and differentiation) correlated to the type of glioma considered.

### 3.3. A2B5 Expression Is a Marker of Cancer Stem Cell (CSC) in Glioblastoma

Around twenty years ago, several studies have reported that human GBM contained cells with NSC features favoring the hypothesis that GBM might arise after their malignant transformation [88,89,90]. Moreover, prominin-1 (CD133) was pointed out as a cell surface CSC marker in GBM since CD133+ (but not CD133-) cells isolated from human GBM were able to initiate GBM when injected in an orthotopic mouse model [91]. Subsequently, Ogden et al. [92] identified A2B5 as a cell surface marker of tumor-initiating cells in adult gliomas. First, they used flow cytometry to demonstrate in 25/25 gliomas the presence of A2B5-expressing cells. Moreover, they showed that the number of A2B5+ cells was much higher than CD133+ cells (respectively, 61.7 +/- 3.8% and 14.8 +/- 3.6%) and observed that all CD133+ cells were A2B5+. Lastly, they showed that both A2B5+/CD133+ cells and A2B5+/CD133- cells were able to generate tumors in nude rats in contrast to A2B5- cells. Therefore, on the basis of the expression of the two cell surface markers A2B5 and CD133, they observed that glioma contained three cell populations: A2B5+/CD133+, A2B5+/CD133- and A2B5-/CD133-. Another study reported a lower proportion of CD133+ cells in three GBM samples (4.6 +/- 1.5%) and confirmed that all CD133+ cells were A2B5+ [87]. By using A2B5 MACS to isolate and characterize A2B5+ and A2B5- cells from human GBM, we showed that A2B5+ cells were able to generate huge and highly infiltrative tumors when implanted into nude mice brains in contrast to A2B5- cells (Figure 4B) [93]. The tumorigenic potential of A2B5- cells was extremely low, since only rare tumor cells were recorded in 2/7 animals implanted with A2B5- cells. Surprisingly, another study reported that A2B5- cells were able to generate tumors in up to 13/16 animals [87]. The discrepancy between this study and the two others [92,93] likely relies on the animal model used: in Auvergne et al.’s study [87], the experiments were conducted in SCID/NOD mice, which offer a much more permissive background than nude rats or mice used in the two other studies [92,93]. We also characterized the functional properties of A2B5+ GBM cells. First, we showed that A2B5+ but not A2B5- cells were able to form numerous and large gliomaspheres in stem cell-permissive medium. In contrast, both A2B5+ and A2B5- cells isolated from human SVZ embryos were able to generate spheres that were much smaller than those derived from A2B5+ cells isolated from human GBM. Then, we observed that the dissociation of primary spheres generated from A2B5+ GBM cells were able to form secondary spheres and limit dilution assay, which showed that the clonal frequency was up to 70%. We also observed that the self-renewing capacity of A2B5+ cells derived from GBM was much higher than those of A2B5+ human embryonic SVZ cells. When placed in a differentiation assay, secondary spheres obtained from A2B5+ GBM cells contained dividing A2B5+ cells and differentiated cells belonging to neural, astrocytic and oligodendroglial lineages, with sometimes aberrant co-expressions of glial and neuronal markers. Then we observed that secondary spheres generated from primary GBM A2B5+ spheres contained the three A2B5+/CD133+, A2B5+/CD133- and A2B5-/CD133- cell populations previously described [92], and that both A2B5+/CD133+, A2B5+/CD133- fractions were tumorigenic in vivo. 

Subsequently, Sun et al. [94] aimed to characterize the invasion properties of A2B5+/CD133- cells (called by the authors “glioma initiating cells—GICs”). To this aim, they first generated gliomaspheres from a human GBM sample, then they used MACS to isolate A2B5+/CD133- (GICs) from the remaining cells (non-GICs). The GICs exhibited a higher invasion capacity in matrigel than the non-GICs. Moreover, GICs demonstrated higher invasion-associated markers including intercellular adhesion molecule-1: ICAM1 (*p* = 0.016), matrix metalloproteinase 2 (MMP2) and MMP9 (*p* < 0.001) than the non-GICs. The expression of the tissue inhibitor of metalloproteinase 3 (TIMP3) was less expressed in GICs in comparison to the non GICs population (*p* = 0.003). At last, the GICs cells displayed a greater invasive capacity than the non-GICs. 

Taken together, these studies recognize A2B5 as a GBM CSC marker and highlight the occurrence of different subpopulations among A2B5+ CSCs with distinct properties.

Since almost all gliomaspheres isolated from fresh human GBM expressed A2B5, we and other have generated CSC GBM cell lines by isolating first A2B5+ cells from fresh human GBM [87,93]. The number of A2B5+ cells is maintained through serial passages and is recorded in almost all cells after the third passage [87]. Besides, the number of CD133+ cells that may vary from one cell line to another remains stable in A2B5+ GBM cell lines. Among the A2B5+ GBM cell lines that we have generated, two of them have been widely studied. They have been obtained from two primary adult GBM located, respectively, near the SVZ (GBM6 cell line) and the cortex (GBM9 cell line). Importantly, these two cell lines displayed different molecular profiles and differential properties in vitro and in vivo [95]. Orthotopic injections in both the SVZ and the cortex of nude mice showed that the migration patterns of GBM6 and GBM9 cell lines mirrored those of adult and fetal normal NSCs, respectively. Genetic analyses showed that GBM6 had a mesenchymal signature, whereas GBM9 had a proneural signature according to Verhaak et al. [96]. 

In contrast to CSCs, commercially available GBM cell lines displaying strong invading capacities (the two most-studied being U87MG and U251MG cell lines) demonstrate a more circumscribed growth pattern and contain a lower number of A2B5-expressing cells (50.25 +/- 3.06% in the U251MG cell line and 17.5 +/- 0.96% in the U87MG cell line) [97]. However, a derived cell line with stemness features called U251MGC1 derived from the U251MG cell line has been produced. This cell line expresses a higher expression of A2B5, enhanced cell growth and higher migration abilities than the parental cell line [97].

### 3.4. A2B5 Cells from Glioblastoma and Normal A2B5 Cells Exhibit Distinct Transcriptomic Signatures

In order to find pathways specifically associated with glioma oncogenesis, Auvergne et al. [87] performed a transcriptional analysis of A2B5+ cells isolated from both low-grade gliomas (LG, grade II) (n = 10) and high-grade (HG, grade III and IV) gliomas and “normal” A2B5 cells derived from four samples of epileptic tissue resections. They identified a set of 355 dysregulated genes (226 up and 113 down). Among those, eight were > 10-fold overexpressed (*MMP3*, *IGFBP3*, *CSRP2*, *SIX1*, *SATB2*, *GAP43*, *EYA1* and *CD24*) and six were 10-fold downregulated (*OPALIN*, *MOBP*, *ANKRD43*, *C21Orf131* and *SPOCK3*). Moreover, in comparison with their “normal” counterparts, glioma A2B5+ cells overexpressed genes involved in TGFβ, BMP and WNT pathways. In comparison to A2B5+ cells isolated from LG gliomas, the A2B5+ cells isolated from HG gliomas were enriched in mesenchymal/epithelial-mesenchymal transition pathways. At last, in comparison with “normal” A2B5+ cells, A2B5+ cells isolated from both LG and HG gliomas were enriched in NSC and OPC markers. Subsequently, they showed that SIX1 silencing inhibited the growth, proliferation and survival of A2B5+ cell lines derived from GBM in vitro and their tumorigenicity in vivo. Later, the same team demonstrated that the pharmacological inhibition of *PAR1* (another gene overexpressed in A2B5+ cells isolated from gliomas) induced a decrease of proliferation and migration of human-derived A2B5+ glioma cell lines derived from GBM in vitro [98]. Lentiviral strategy was used to knockdown PAR1 in A2B5+ GBM cell lines. Lentiviral shRNAi knocked down of PAR1 in two A2B5+ cell lines derived from GBM significantly reduced the expansion and self-renewal of the cell line in vitro. Moreover, mice implanted with A2B5+ *PAR1* knockdown had an increase in overall survival in comparison to the A2B5+ *PAR1*sh control GBM cell lines or to the parental A2B5+ GBM cell lines [98].

### 3.5. A2B5 Cells in Glioblastoma Change Their Phenotype in Response to Environmental Clues

In addition to CD133 and A2B5, other cell surface markers have been found in GBM cells with CSC properties. These include CD15, CD44, CXCR4, ITGA6, L1CAM and CD36 [99,100,101,102,103,104,105]. These findings suggest that a large variety of phenotypic CSC occurs in GBM. Moreover, by studying the expression of these eight cell surface markers by flow cytometry in gliomaspheres obtained from various cell lines including U87MG, U251MG and five other GBM stem-like cell lines purchased from Cell Line Services (CLS, Eppelheim, Germany NCH421K and NCH64436) or derived from GBM patients from a phase II clinical trial (NCT01213407 Linz1, Linz2 and Gli16), it was shown that all gliomaspheres expressed at least a combination of five different cell surface GBM CSC markers, whereas two cell lines (NCH421K and NCH644) expressed the eight selected surface molecules [106]. Although the differences in cell surface CSC markers from one cell line to another are in keeping with the great heterogeneity of GBM, it is obvious that changes in cell surface CSC markers occur within each GBM and depend on both intrinsic (clonal expansion) or extrinsic factors (microenvironment) and reflect the high plasticity of glioma CSCs. Several important studies have confirmed these hypotheses by using, among others, the A2B5 cell surface marker [107,108,109]. Differences in CSC cell surface markers including A2B5 were shown in distinct clones derived from parental GBM [107]. By combining ploidy-based flow sorting with array comparative genomic hybridization, it was shown that GBM can be classified into two main groups: monogenomic (containing a pseudodiploïd clone mixed with normal stromal cells) or polygenomic (containing multiple tumor clones, one of them being pseudodiploïd leading to aneuploïdy). In this last group, aneuploïdy can be regarded as a late event in GBM evolution since pseudodiploïd and aneuploïd fractions carry the same genetic aberrations defined by identical chromosomal breakpoint. The expression of CSC cell surface markers including A2B5 was analyzed by flow cytometry in monogenic GBM xenografts and in two polygenomic tumors. In monogenic tumors, the number of A2B5+ cells ranged from 0 to 100% depending on the tumor analyzed, reflecting inter-tumoral heterogeneity. In polygenic tumors, the number of A2B5+ cells was different in the pseudodiploïd fraction *versus* the aneuploïd one: for example, 21% *versus* 2.8% in one tumor and 78 *versus* 60% in another one. More recently, the same team further demonstrated that the phenotypic heterogeneity based on the expression of CSC cell surface markers including A2B5 was a dynamic process of reversible state transitions [108]. Changes in the expression of CSC surface markers is not an intrinsic property of emerging clones with specific genetic alterations but rather an adaptation process to external clues such as changes in oxygen content [108]. Interestingly, other authors set up an original technology combining a barcode labelling approach and CSC cell surface markers detection, including A2B5, CD44, CD15 and CD133, to study the phenotypic plasticity of the cell surface markers in response to changes in the environment in GBM clonal populations [109]. Although all these studies demonstrated that GBM cell surface expression is a highly dynamic process, they recognized A2B5 as a powerful surface marker to study CSCs dynamics.

### 3.6. Generation and Functional Properties of the A2B5 Epitope in Glioblastoma Cells 

The above paragraphs unambiguously showed that A2B5-expressing cells in GBM have CSC properties. Data also showed that A2B5 expression at the cell surfaces of glioma cells in a given GBM also depends on intrinsic genetic differences among clones or external clues. However, none were giving insights on the functional properties conferred by its expression on the GBM cell surface. To this aim, we have first demonstrated that the expression of the enzyme ST8SIA3 (see paragraph 1) was correlated with A2B5 immunostaining [110]. The *ST8SIA3* gene was stably overexpressed by lentiviral infection or silenced by using shRNA technology in two GBM cell lines expressing mild (U251MG, 50.25 ± 3.06%) and low (U87MG, 17.5 ± 0.96%) levels of A2B5 immunoreactivity. ST8SIA3 mRNA was significantly increased in ST8SIA3-overexpressing cells in comparison to the wild-type cell lines and the sh-cell lines. We observed that the overexpression of A2B5 in ST8SIA3 cell lines was associated with increased cell proliferation, clonogenicity and migration in GBM in vitro and tumorigenicity in vivo. Conversely, lentiviral ST8SIA3 inactivation in low-A2B5-expressing cells resulted in reduced proliferation, migration and clonogenicity in vitro and extended survival when grafted in mice. The lentiviral delivery of shST8SIA3 in CSC cell lines (GBM6 and GBM9) expressing a high level of A2B5 stopped their growth. As an alternative, we used neuraminidase treatment to remove the A2B5 epitope at the cell surface and observed impaired cell survival, proliferation, self-renewal and migration. Altogether, these observations support the view that the A2B5 epitope plays a crucial functional role in the promotion of proliferation, migration, clonogenicity and tumorigenesis of GBM cells. 

## 4. Conclusions

A2B5 is a marker of cells with glial-restricted/neural stem cell properties both in the CNS and in gliomas.

It is expressed by distinct neural precursor cells depending on the developmental stage. During development, A2B5 not only characterizes OPCs, but it is also expressed by a subset of radial glial cells. In adulthood, A2B5 is still found in neurogenic niches SVZ and SGZ, and it also identifies OPCs and multipotent neural progenitor cells from subcortical white matter. Nevertheless, mapping precisely A2B5 expression remains an issue. A2B5 is an IgM and does not properly work after fixation on tissue, resulting in some gaps in the characterization of its expression mostly due to the use of NG2 and PDGFRα to characterize OPCs. Deciphering whether A2B5 could be expressed by the GFAP+ NSCs of the SVZ would be crucial information in favor of A2B5+ cells as candidates to be at the origin of gliomas.

We have shown that A2B5 expression is shared by all gliomas whatever their types and grades. In GBM, A2B5+ expressing cells have CSC properties, and A2B5 is one of the most powerful cell surface markers to recognize these CSCs. ST8Sia3 is crucial to generate A2B5 expression in GBM cells and therefore to participate in the control of their biological properties. Although the high plasticity of CSCs might limit therapeutic strategies targeting CSC cell membrane components to cure GBM, it remains that A2B5 is an attractive glioma therapeutic target because of its widespread expression. To this aim, several therapeutic strategies can be proposed: targeting pathways activated in glioma cells expressing A2B5 [87], targeting the expression of the A2B5 epitope, or targeting A2B5-expressing cells by immunotherapy.

Two main strategies might be instrumental to target the A2B5 epitope: silencing ST8Sia3, the enzyme leading to A2B5 synthesis on GD3 ganglioside by a small interfering (si) RNA approach, or removing the sialic acids of the A2B5 ganglioside by using neuraminidase. Although siST8Sia3 RNA has never been reported so far, this strategy has been successfully used to inhibit ST8Sia1, also called GD3 synthase, in lung cancer experimental models [111]. It is obvious also that targeting GD3, the precursor of GT3, the main A2B5 carrier, will subsequently abrogate A2B5 expression.

The suppression of A2B5 epitope by neuraminidase is an option shown to be instrumental in some preclinical studies dedicated to the treatment of neurologic disorders such as spinal cord injury or hyperalgia in peripheral-neuropathy induced by type 1 diabetes [112,113]. Although a long way remains to use neuraminidase treatment in human disorders, including glioma treatment, these two examples show the feasibility of this approach. 

At last, various immunotherapy approaches might be used to eliminate A2B5+ cells, mainly including dendritic cell therapies and chimeric antigen-receptor T cells (CAR-T cells). In a preclinical setting, a mouse glioma immunotherapy mediated by A2B5+GL261 (a syngeneic mouse model of GBM) cell lysate-pulsed dendritic cells (DCs) was set up [114]. In this model, the vaccination with A2B5+GL261 lysate-pulsed DCs prevented effects against glioma formation but had no longer an effect when the tumors were formed. Interestingly, a phase II study is in progress (NCT01567202) in a single center to determine the efficacy of autologous DCs loaded with autogeneic glioma stem-like cells (A2B5+) administered as a vaccination in adults with GBM. Although CAR-T cells against A2B5 have not been achieved yet, a CAR-T cell was successfully designed and used to target the ganglioside GD2 in a mouse model of H3K27-mutated midline gliomas [115]. An adjuvant monoclonal antibody, 8B6, which targets O-acetyl GD2, which impairs temozolomide resistance driven by glioma CSCs, was also reported [116]. 

To conclude, the A2B5 epitope, which is carried by complex gangliosides, plays important functions in the development of the nervous system and in gliomas. However, in the normal developing and adult CNS, it is difficult to reconcile A2B5 expression with the recent literature focusing on OPCs that do not use this marker. Nevertheless, A2B5 can be considered a powerful cell surface marker of OPCs and glioma cells, and in GBM, A2B5-expressing cells have CSC properties. Altogether A2B5 is an attractive glioma therapeutic target.

## Data Availability

Not applicable.
